# ComiR: combinatorial microRNA target prediction tool

**DOI:** 10.1093/nar/gkt379

**Published:** 2013-05-22

**Authors:** Claudia Coronnello, Panayiotis V. Benos

**Affiliations:** ^1^Fondazione Ri.MED, 90133 Palermo, Italy, ^2^Institute of Biomedicine and Molecular Immunology “Alberto Monroy” IBIM, National Research Council, 90146 Palermo, Italy and ^3^Department of Computational and System Biology, University of Pittsburgh School of Medicine, 15260 PA, USA

## Abstract

ComiR is a web tool for combinatorial microRNA (miRNA) target prediction. Given an messenger RNA (mRNA) in human, mouse, fly or worm genomes, ComiR computes the potential of being targeted by a set of miRNAs, each of which can have zero, one or more targets on its 3′untranslated region. In determining the regulatory potential of an mRNA from a set of miRNAs, ComiR uses user-provided miRNA expression levels in a combination of appropriate thermodynamic modeling and machine learning techniques to make more accurate predictions. For each gene, ComiR returns the probability of being a functional target of a set of miRNAs, which depends on the relative miRNA expression levels. The tool provides a user-friendly interface to input a miRNA expression table containing many sample information and filter out the most relevant miRNAs. ComiR results can be downloaded or visualized on a table, which can then be used to select the most relevant targets and to compare the results obtained with different miRNA expression input. ComiR is freely available for academic use at http://www.benoslab.pitt.edu/comir/.

## INTRODUCTION

MicroRNAs (miRNA) are a class of short (18–25 nucleotide) non-coding RNAs that regulate gene expression post-transcriptionally. Their regulatory activity depends heavily on the recognition of binding sites located mainly on the 3′-untranslated regions (3′UTRs) of target messenger RNA (mRNA) (1). Existing computational tools predict the miRNA targets by considering site-specific factors of target sites [see (2–4) for some examples]. These tools, tested against the available experimentally validated miRNA-target pairs each, were able to predict only a portion of them, while their target overlap remains poor (5,6). However, the efficiency of miRNA-mediated regulation can be affected by multiple system-wide factors (7,8), such as miRNA expression, combinatorial binding of multiple miRNA targets or the expression of competitive endogenous RNA. ComiR addresses two of them, namely how miRNA expression affects binding and how combinatorial miRNA binding affects mRNA regulation. We note that some recent algorithms also consider these factors when they decide on targets: PicTar (9) is a computational method for identifying common targets of miRNAs, but it does not consider the miRNA expression in determining the relative binding; GenMir++ (10), TargetMiner (11) and TaLasso (12) evaluate the relevance of miRNA:mRNA interactions by analyzing expression profiles and prior targeting information, but they require paired mRNA and miRNA expression profiles over many samples. Furthermore, none of them incorporates miRNA expression in the binding model.

Recent experimental approaches, like those based on immunoprecipitation (IP) of miRISC proteins (RNA-induced silencing complex) (13,14), generate validated miRNA:mRNA target data in a high-throughput fashion. These data are useful to unravel the systemic aspects of miRNA targeting. Indeed, miRNA expression profiles are now known in several tissues and cell lines (15) and as a result it becomes more important to know the targets of a set of miRNA genes instead of individual miRNAs. Of course, one might infer the targets of a set from the targets of its members, but as we have recently shown, a naïve inference model does not produce good results (16).

To this end, we developed ComiR (http://www.benoslab.pitt.edu/comir/), an algorithm that predicts the regulatory potential of a set of miRNA genes toward a given mRNA. ComiR initially integrates miRNA expression in a scoring scheme for each single target on this mRNA, and then it additively combines the weighted scores of the single targets. ComiR uses four popular scoring schemes [the ones used by miRanda (4), PITA (2), TargetScan (3) and mirSVR (17)] and at the final stage it combines all four integrative scorings into one using a support vector machine (SVM) trained on *Drosophila* AGO1 IP data (18). The end result is the SVM probability, which represents the likelihood that this mRNA is regulated by the set of miRNA genes. Our results (16) have shown that ComiR presents a significant improvement over standard miRNA prediction algorithms adapted for combinatorial miRNA targeting.

## TOOL DESCRIPTION

### General framework

ComiR is a tool designed to predict the targets of a set of miRNAs by considering the miRNA expression levels as an integral part of the target decision process. The user has the option to add his/her own target set of genes (e.g. 3′UTRs of mRNAs, LINCs) to the default list of genes considered for the available species (*Homo sapiens*, *Mus musculus*, *Drosophila melanogaster* and *Caenorhabditis elegans*) Below, without loss of generality, references to ‘target sequences’ or ‘mRNAs’ should include the 3′UTRs of the mRNAs together with any other custom target sequence set the user submits.

For each mRNA, ComiR determines the potential of being targeted by the miRNA set. The targeting potential is calculated in two steps. In the first step, four different methods are used that complement each other to some extent. One method is an adaptation of miRanda (4), in which the probability of an mRNA:miRNA binding is calculated based on the Fermi–Dirac equation (16,19), which takes into consideration the miRNA expression, and the individual probabilities are summed over all targets on this mRNA of all miRNAs in the miRNA set. The second method is a similar adaptation of PITA (2), in which the Fermi–Dirac equation also substitutes the standard energies. As the third method, we use the TargetScan (3) scoring (without conservation) weighted by each miRNA expression level. Finally, mirSVR (20) is also used, but its scores are also combined after they have been weighted by miRNA expression. In the second step, we combine the predictions of the above four methods with a SVM, which is trained on a high-quality data set derived from AGO1 IP experiments in *D.melanogaster*. It has been shown (16) that this model can be efficiently applied to predict miRNA targets in other species, after a normalization step.

**Figure 1.**
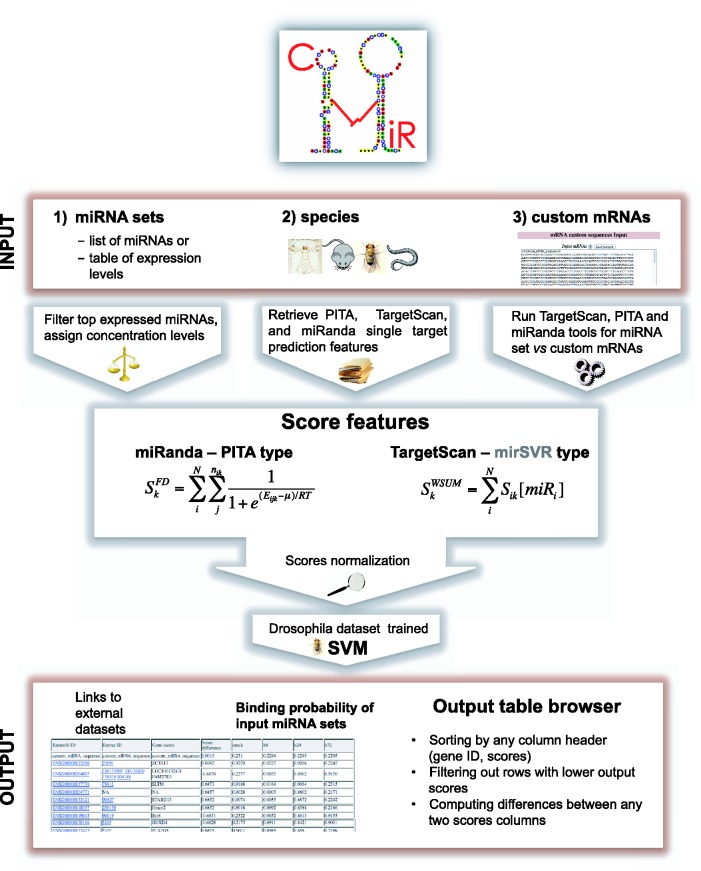
Overview of the integrated analysis in ComiR.

ComiR’s output is a ranked list of genes based on the target probability computed through the SVM model. Because the score computation includes the miRNAs expression values, the same set of miRNAs might generate different ranking if different miRNA expression levels are used. ComiR’s pipeline is shown in Figure 1.

### Databases and pre-computed binding scores

The ComiR help page provides links to the FASTA files with the mRNA and miRNA sequences used to run ComiR. We restricted our analysis on the mRNA 3′UTR sequences that we downloaded from the ENSEMBL Web site (as in 20 September 2012). When more than one 3′UTR sequence is associated to the same ENSEMBL gene ID, we use the longest sequence. miRNAs mature sequences were downloaded from the miRBase Web site (Release 19). An internal ID conversion tool is used to convert the old miRNA IDs with the updated ones. Regardless, we suggest the users to refer to the latest version of miRBase official ID.

miRNA binding scores have been pre-computed for every 3′UTR gene sequence according to the original miRanda, PITA, TargetScan and mirSVR scoring systems and they are stored on our server for a fast access. PITA and TargetScan ran with their default parameters. The miRanda’s parameters have been relaxed to include in the predictions the weaker binding sites (score = 0, energy = 0). Finally, we used the mirSVR prediction files labeled as ‘Good mirSVR score, Conserved miRNA’. The single target scores we use as basis to calculate the overall regulatory potential of each miRNA:mRNA pair are the following. (i) miRanda-type scoring: single target score is the binding energy of each binding site. (ii) PITA-type scoring: single target score is the difference between the free energy gained by the binding of the miRNA to the target and the free energy lost by unpairing the target-site nucleotides (ΔΔG) of each binding site. (iii) TargetScan-type scoring: single target score is the number of detected binding sites (no phylogenetic conservation features are used). (iv) mirSVR scoring: single target score is mirSVR score of the predicted target sites.

### Input formats

ComiR accepts miRNA expression levels input as comma-separated field table format. The first row contains the sample IDs where miRNA expression levels are detected and the first column contains miRNA IDs. If miRNA expression levels are expressed in log scale (e.g. from microarray measurements), ComiR will internally convert them into the linear scale, provided the user checks the corresponding checkbox. We also allow users to submit a list of miRNA IDs without expression levels, in which case ComiR assumes that all miRNAs are expressed at the same level. Custom mRNA sequences can be inserted with FASTA file format.

### Data pre-processing

The input miRNA IDs are first converted into the latest miRBase IDs. The miRNAs that are not found in the stored data set related to the selected species are removed from the analysis and the user is notified. If miRNA expression levels are provided, the miRNA concentrations are computed as the fraction of expression level over the total amount, for each sample. It is possible to focus the analysis on the top expressed miRNA, by selecting the percentage of total miRNA abundance to be covered in the ‘More Options’ panel, and/or a threshold for the minimum expression level to be considered. It is also possible to input a list of miRNAs to be excluded from the filtering, even if their expression level does not reach the desired threshold.

The list of miRNAs can be treated in two different ways, i.e. as a set of miRNAs or as single miRNAs. One of the two options can be selected in the ‘More Options’ panel. If the user wants the miRNAs in the list to be considered as a set, ComiR will compute one score for each gene. For multiple samples, ComiR will calculate one score per gene per sample. If the user wants each miRNA in the list to be considered independently, ComiR will compute one set of scores per miRNA, by using a concentration equal to 1 for each of them even if the miRNA input contains expression levels.

If custom target sequences are provided, ComiR will run the publicly available scripts of miRanda, PITA and TargetScan tools, to obtain the initial binding energy and target prediction scores and these values will be transformed and combined as we describe above.

### Score combination and method integration

As we mentioned above, ComiR uses four complementary methods to assess the binding potential of a miRNA to a single target in each 3′UTR. One is based on simple binding energies, which we take from the miRanda scoring file. The second is based on binding energies after the mRNA secondary structure has been factored in. For this we use the PITA scoring file. The third is the number of string matches of the miRNA seed sequence, which we take from the TargetScan primary predictions. And the fourth method is the prediction score of mirSVR. ComiR transforms these primary scores of individual targets using the miRNA expression and sums the transformed scores to account for multiple miRNA targets. The summing is a simple weighted sum in the case of TargetScan and mirSVR types of scores, whereas in the case of miRanda and PITA types we use the Fermi–Dirac equation. The details are provided in (16). Briefly, the Fermi–Dirac model is the following:

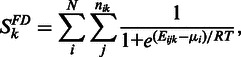

where 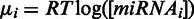, *N* is the number of miRNAs in the input set, *n_ik_* is the number of binding sites predicted for the miRNA_i_:mRNA_k_ pair and for each of them *E_ijk_* is the binding energy computed for the binding site *j*. For the seed matching scores and the mirSVR scores we use a simpler scoring scheme (weighted sum):

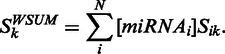



At the end of this step, each gene is associated with four scores, one for each primary method. These four scores are then used as input to the SVM model, which has been calculated on a *D.melanogaster* AGO1 IP data set (16). To use the SVM to efficiently predict targets in other species, the algorithm includes a normalization step. Specifically, the input to the SVM is the ranking of the genes with respect to the computed combined scores. This expedient avoids the biases due to different prediction scores distributions in different species and to the variety of miRNAs the user may use as input. Finally, the SVM computed probability is chosen as the ComiR score referring to the probability of an mRNA to be regulated by the set of miRNAs with the specific expression levels.

### Submission and wait time

Depending on the size of the input files and the chosen species, the analysis could take anywhere from seconds to up to an hour. As an example, for *D.melanogaster* predictions with 25 miRNAs, the computing time is roughly 25 s, while the same number of miRNAs in *H.sapiens* predictions is processed in roughly 5 min. The inclusion of custom mRNA sequences will increase the execution time on the server linearly with the number and the length of the inserted sequences. For instance, processing one custom sequence of 3000 bases and 25 miRNAs requires about 45 extra seconds.

After submission, a summary page describing the input and the results of the data pre-processing is shown. If the input setting needs to be adjusted then a ‘Go to Input settings’ button leads back to the input page. Otherwise the ‘Go to Results’ button brings to an execution log page. This page will contain the results once the job finishes and the link to it can be saved so it can be visited later.

### ComiR output

The user can decide to download the complete results in a table format or to browse and filter the results table in the web page. The downloadable results zip file contains the Ensembl ID and the Official Gene ID columns followed by the computed ComiR scores columns, one per each miRNA set present in the miRNA input file. If custom mRNA sequences have been inserted, the file will also contain a row for each inserted sequence.

The results web page shows a table with the first three columns describing the genes followed by the computed ComiR score columns. The genes are identified by the Ensembl ID, Entrez ID and Official Gene Name. Ensembl IDs and Entrez IDs are linked to the correspondent external database. The user can sort the table rows by any header and in ascending or descending order. It is possible to modify the shown results table by removing all the genes not predicted as targets by any of the input miRNA sets, by choosing the ComiR score threshold above which the genes has to be considered as targets. Finally, if more than one miRNA set is inserted, it is possible to visualize an extra column containing the difference between the ComiR scores obtained with any two miRNA sets. Moreover, if enough data are available, it is possible to perform a Wilcoxon analysis comparing the ranks of the genes in ComiR predictions of two groups of at least three miRNA sets. In fact, it is possible to select the composition of the two groups of samples to be compared and a new column containing the Wilcoxon-test *P*-value will be shown. In this case, the score difference column will contain the difference between the average ComiR scores in the two groups. The genes with significant *P*-values are what we call the ‘differentially predicted’ genes. The results visualization and the differentially predicted genes analysis can also be performed by considering the rank of the genes with respect of the associated ComiR score instead of the ComiR score itself.

## CASE STUDY SCENARIOS

The primary goal of ComiR is to rank the targets of a set of miRNAs with known expression levels in the sample of interest. In (16) we described some applications of ComiR in this basic direction, where we predicted the targets of *D.melanogaster*, *C.elegans* and *H.sapiens* miRNA sets and showed that ComiR outperforms existing target prediction tools. Moreover, ComiR can be applied to perform more elaborate analysis, and some examples are described above.

### Single-nucleotide polymorphisms effects on miRNA binding

Single-nucleotide polymorphisms (SNP) can be located in miRNA binding regions and consequently they could affect gene expression. ComiR can be used to evaluate the SNP’s effect on miRNA regulation by inserting as input the wild type and the SNP mRNA sequence and ranking the evaluated ComiR scores. As an example, in (16) we evaluated the effect of 15 high-frequency SNPs in ERα pathway genes on the affinity of the affected genes with every known human miRNA. We found that rs17737058, located in the 3′UTR of NCOA1 gene, causes the disruption of the binding with the hsa-miR-488* miRNA. This effect has been experimentally validated, and we also found that this SNP is associated with decreased bone mineral density.

### Differentially predicted genes

This analysis is thought to compare the target predictions obtained with different miRNA sets. It is, for instance, conceived for data sets containing replicates of miRNA expression levels data in different samples. The differentially predicted genes analysis can be applied as an alternative strategy to predicting the targets of differentially expressed miRNAs. In fact, with the same list of miRNAs, ComiR generates different results, depending on the concentrations associated with the miRNAs. It is worth noticing that while predicting the targets of the differentially expressed miRNAs does not take into account the presence of all the other miRNAs that might prevail over them, ComiR can be used to predict the targets of the top-expressed miRNAs in each sample, and consequently used to search for which genes are highly predicted in one group of samples and not in the other (21).

### Single miRNA contribution

ComiR algorithm incorporates the information about all the input miRNAs into a single score. If the user is interested in determining which miRNAs contributed more to the formation of one specific gene’s score, we suggest to run ComiR with the same miRNA set, but with the option ‘single miRNAs’ activated. This analysis will run a separated ComiR analysis for each miRNA in the set. The higher is the contribution of a specific miRNA to the ComiR score associated to the entire miRNA set, the higher will be the ComiR score associated to the single miRNA.

## CONCLUSIONS

In recent years, with the availability of high-throughput miRNA expression data, a new perspective for miRNA target prediction is starting to emerge. Many target prediction tools already exist, but none of them takes into account system-level factors, like the co-expression of several miRNAs with sample-specific expression levels. ComiR has been developed specifically to predict the targets of sets of miRNAs, and its user-friendly interface allows researchers to compare the results obtained with groups of them. A number of issues remain unsolved, however, such as the competition of miRNAs for the Ago proteins (22), or the extent of the co-operative effect of miRNAs. As more relevant data sets become available we will extend the miRNA binding models to incorporate these effects.

## FUNDING

National Institutes of Health (NIH) - National Library of Medicine (NLM) R01 [LM007994 and LM009657 to P.V.B.]; Fondazione Ri.MED (to C.C.). Funding for open access charge: NIH - NLM R01 [LM009657].

*Conflict of interest statement*. None declared.
